# Development, reliability, and validity of a self‐assessment scale for dementia care management

**DOI:** 10.1111/psyg.12937

**Published:** 2023-02-01

**Authors:** Kana Kazawa, Mariko Mochizuki, Hiroyuki Ochikubo, Shinya Ishii

**Affiliations:** ^1^ Department of Medicine for Integrated Approach to Social Inclusion Graduate School of Biomedical and Health Sciences, Hiroshima University Hiroshima Japan; ^2^ Hiroshima Care Manager Association Hiroshima Japan

**Keywords:** dementia, long‐term care, self‐assessment, validation study

## Abstract

**Background:**

This study aimed to develop a self‐assessment scale for care management of people with dementia and examine its reliability and validity.

**Methods:**

Based on Bloom's Taxonomy, previous research, and experts' opinions on dementia and care management, a scale consisting of 18 items was developed to assess care managers' attitudes, knowledge, and skills in their management of people with dementia. To examine the scale's reliability and validity, data were collected from 638 care managers. Construct validity using exploratory factor analysis, known‐group validity, and internal consistency reliability of the scale were evaluated.

**Results:**

Exploratory factor analysis supported the construct validity with a four‐factor model and explained 59.1% of the total variance. Following were the four factors: Factor I ‘Person centred care’; Factor II ‘Understanding of disease characteristics, treatment and care’; Factor III ‘Understanding of people with dementia and care management according to their characteristics’; and Factor IV ‘Utilization of local resources surrounding people with dementia’. Regarding the known‐group validity, results showed that the group with a qualified chief care manager scored significantly higher than the group without one on Factors I (*P* = 0.013) and III (*P* = 0.026). Cronbach's alpha coefficient for the 18 items was 0.928.

**Conclusions:**

The findings prove that the scale has acceptable reliability and validity, and can help care managers reflect on their practice. Future research is desirable to measure the validation of change in the scale.

## INTRODUCTION

With the population ageing worldwide, in Japan the percentage of older people ≥65 years in 2021 was reported to be 28.8% of the total population.^1^, ^2^ As medical technology advances and the population ages, the number of older people with multiple chronic conditions that are difficult to cure, such as dementia, or who require daily long‐term care, has increased. In Japan, 2.9% of the older population aged 65–74 and 23.0% of those aged ≥75 need long‐term care,^2^ and about 55.0% of these need care due to dementia.^3^ Furthermore, a survey of people with early‐onset dementia reported that 70% of respondents were certified as requiring care under the long‐term care insurance system.^4^

When caring for people with dementia who require long‐term care, person‐centred care that emphasizes the perspective of the person with dementia, builds a mutually trusting relationship, and enables the person to express their personality and abilities is fundamental.^5^, ^6^ Care providers are required to manage the disease, support daily living, and support decision‐making regarding these issues to improve quality of life, based on a dementia‐centred approach.

In Japan, the long‐term care insurance system was introduced in 2000. It was based on the principle that society as a whole should provide support by responding to the increasing number of people and those requiring long‐term care.^7^ Under this system, it is the care manager who is responsible for assessing the care needs of those requiring long‐term care, and for planning and coordinating their individual care plans.^8^, ^9^ One care manager is assigned to each person certified as needing long‐term care, and most payments for care managers are made from the long‐term care fees.^10^ The basic qualifications of care managers are experience as professionals in medical and long‐term care‐related occupations such as doctors, nurses, physical therapists, occupational therapists, case workers, and social workers. To ensure the quality of dementia care, the Long‐Term Care Insurance Law requires the care managers to receive regular education on dementia, dementia care, social security systems including long‐term care insurance, and community resources.^11^ Therefore, to further maintain and improve the quality of their care management practices, the perspective of evaluation is necessary. An evaluation indicator is useful for care managers to reflect on their practice, and this can lead to continuous quality improvement of their care management.

Research on quality assessment of care for people with dementia based on person‐centred dementia care has been developed for a variety of settings. In institutional settings, nursing practice evaluation in acute care hospitals^12^ and care process evaluation in dementia group homes^13^ have been reported. In communities, quality evaluation through a joint team approach between healthcare professionals and informal caregivers has been reported.^14^ Further, evaluation of staff competencies at dementia cafes where people with dementia, their families, local residents, and healthcare professionals mutually share information and understand each other has been reported.^15^ In contrast, there is no research that attempts evaluation focusing on the expertise of care managers who collaborate with multiple professions, and evaluate and coordinate people with dementia‐centred medical and long‐term care services.

This study developed a self‐assessment scale for care managers of people with dementia, and examined its reliability and validity.

## METHODS

This study adhered to the COSMIN reporting guideline for studies on measurement properties of patient‐reported outcome measures.^16^

### Development of a self‐assessment scale for care management of people with dementia

Since the competencies required for care management for people with dementia are lifelong learning and study, Bloom's Taxonomy^17^ was consulted as the structural basis for this scale.

Based on previous research,^12^, ^13^, ^14^, ^15^ 18 questions were developed to examine the components of the scale, under topics including ‘Person‐centred care’, ‘Understanding of disease characteristics, treatment and care’, and ‘Care management according to the individual characteristics of people with dementia’ (see Supplemental File 1 in Data **S1**, in the online Supporting Information, for the 18 questions in Japanese).

To confirm the surface validity of the created scale, discussions were held with a dementia specialist, a care management specialist, and an expert in dementia care to obtain their opinions on the validity of the scale. Based on the results of these discussions, the 18 questions were adopted. 

### Evaluation of reliability and validity of the scale

#### Participant selection

The care managers who belonged to the Hiroshima Care Manager Association during October 2021 were solicited as participants for the evaluation of the scale's reliability and validity. At the time of the survey, 2080 persons were registered with the Hiroshima Care Manager Association and were provided with self‐reporting questionnaires through the association. Of these, 638 responded to the survey anonymously and were included in the study (response rate: 30.7%).

#### Data collection

The questionnaire consisted primarily of the 18 item self assessment scale for dementia care management. Participants answered each item on a 4‐point scale (4 = Applies, 3 = Somewhat applies, 2 = Somewhat does not apply, and 1 = Does not apply), with higher scores indicating a higher self‐evaluation of care management. Data regarding age, gender, medical, and long‐term care‐related qualifications of the participants, years of experience as a care manager, and whether or not the participant worked as a chief care manager were collected. The chief care managers are certified after 5 years of experience as a care manager or 3 years of experience as a care manager and specialized training. They have sufficient knowledge and experience regarding care management and are also involved in care manager education. It was reported that in long‐term care insurance facilities that had a certain number of chief care managers, the risk of deterioration in the level of long‐term care required by users who received care management from the chief care managers tended to decrease.^18^ Therefore, as a variable of interest for this study, it was considered that certified chief care managers provided better care management practices for people with dementia.

#### Statistical analysis

First, descriptive statistical analysis was conducted.

Next, after removing the missing values, the scale was validated using the following process. Item analysis check for the existence of ceiling and floor effects for each item. The ceiling effect was defined as mean + standard deviation >4 (maximum possible score), and the floor effect was defined as mean – standard deviation <1 (minimum possible score), and items falling under these categories were to be removed. Inter‐item correlations and item‐total correlations were evaluated using Spearman's rank correlation coefficient. A coefficient of ≥0.9 between the items and item‐total correlation coefficients of <0.4 were to be used as criteria for exclusion. Construct validity was evaluated by exploratory factor analysis using maximum likelihood method and promax rotation, and criterion validity was evaluated by the known‐group method. Exploratory factor analysis is widely used to measure variables that cannot be assessed directly and refine a scale based on the results of factor loadings. In this study, a factor loading of ≥0.35 was used as the criterion for adoption. We also confirmed the scale with parallel analysis and conceptual meaningfulness.^19^ Oblique rotation (promax rotation) was chosen as the components/factors were assumed to be correlated.^20^ Known‐groups validity is evidenced when a test can discriminate between two groups that are known to differ in terms of a variable of interest.^21^ Therefore, a *t*‐test was used to compare the scale scores of those with versus those without certified chief care manager status, to ascertain whether these two groups (certified status group, non‐certified group) could be identified based on statistically significant differences in their scores. To examine reliability, Pearson's correlation coefficients were calculated among the subscales employed, and Cronbach's alpha was calculated for the entire scale and for each subscale to verify internal consistency.

### Ethical considerations

Since this study was an anonymous survey and the participants were not identified, approval by an ethics review committee was not required. The questionnaire was distributed to the participants along with an explanatory document describing the purpose, content, and significance of the study, protection of personal information, freedom of participation, and the fact that they could not be identified, and therefore could not withdraw after the consent was provided. The submission of the questionnaire was regarded as consent to participate.

## RESULTS

### Characteristics of the participants

Table 1 shows the characteristics of the participants (*n* = 638). Four hundred thirty‐eight respondents (68.7%) were aged in their 40s or 50s, followed by those aged ≥60 (*n* = 151, 23.7%) and those in their 20s and 30s (*n* = 46, 7.2%). Women comprised more than 70% of the participants, and 358 (56.1%) had >10 years of experience as a care manager. One hundred seventy‐eight were medically qualified (27.9%), and included nurses, public health nurses, pharmacists, and physicians. Three hundred eighty‐nine (61.0%) were certified as a chief care manager.

**Table 1:** Characteristics of the participants

Attributes	*n* (%)
Age	
20s–30s	46 (7.2%)
40s–50s	438 (68.7%)
Over 60	151 (23.7%)
No response	3 (0.5%)
Gender	
Woman	494 (77.4%)
Man	134 (21.0%)
No response	10 (1.6%)
Years of experience as a care manager	
<5 years	120 (18.8%)
5–10 years	156 (24.5%)
Over 10 years	358 (56.1%)
No response	4 (0.6%)
Having a medical qualification	
Yes	178 (27.9%)
Being a certified chief care manager	
Yes	389 (61.0%)
No response	5 (0.8%)

For the item and factor analysis, 611 (95.8%) of those who responded to all 18 items of the scale were included.

### Validity and reliability of the scale

Descriptive statistics (mean and standard deviation) and item‐total correlation coefficients for each item are shown in Table 2. No items showed ceiling or floor effects, and no item‐total correlation coefficients exceeded 0.8. Item‐total correlation coefficients all showed significant correlations (*P* < 0.01), with correlation coefficients ranging from 0.546 to 0.717. Therefore, all 18 items were used in this scale.

**Table 2:** Item analysis of the scale

	Items	Mean	(SD)	Floor effects	Ceiling effects	Item‐total correlation coefficients^†^
				Mean – SD	Mean ± SD	
Q1.	For behaviours related to cognitive impairment, I try to understand the reasons for the behaviour from the perspective of the person with dementia first.	3.37	(0.60)	2.77	3.98	0.652**
Q2.	I try to support people with dementia so that they themselves feel that ‘they can do something’.	3.39	(0.57)	2.82	3.96	0.683**
Q3.	I try to respect the individuality of each people with dementia and support them according to their cognitive function and their own needs.	3.35	(0.57)	2.77	3.92	0.671**
Q4.	Regardless of the level of dementia symptoms, I try to support people with dementia by respecting their wishes and values.	3.31	(0.62)	2.69	3.92	0.652v
Q5.	I try to understand the individual needs and concerns of people with dementia.	3.40	(0.56)	2.84	3.95	0.675**
Q6.	I always consider what is a priority for the person with dementia while anticipating possible unfavourable issues such as falls.	3.32	(0.61)	2.71	3.93	0.626**
Q7.	I understand the core features of dementia and the care required for it.	3.04	(0.57)	2.47	3.62	0.709**
Q8.	I understand the behavioural and psychological symptoms and the care required for it.	3.01	(0.59)	2.42	3.60	0.694**
Q9.	I understand the symptoms and care required at each stage of dementia progression.	2.89	(0.62)	2.27	3.51	0.701**
Q10.	I understand the concepts, characteristics, and treatment of diseases that cause dementia.	2.79	(0.63)	2.16	3.43	0.670**
Q11.	I understand the various physical, psychological, and social needs that arise for people with dementia and their families.	2.98	(0.60)	2.38	3.59	0.675**
Q12.	I understand the adult guardianship system, driver's licence return, and other systems related to dementia.	2.77	(0.71)	2.06	3.48	0.546**
Q13.	I assess the thoughts of people with dementia and their families, their physical and mental condition, and the care provided by the families.	3.12	(0.56)	2.56	3.68	0.653**
Q14.	Based on the information I possess, I consider the care needed and its prioritization for the person with dementia.	3.10	(0.63)	2.47	3.73	0.717**
Q15.	As a care manager, I try to understand the feelings and thoughts of people with dementia, their families, and supporters.	3.23	(0.60)	2.62	3.83	0.677**
Q16.	I establish a system for cooperation with neighbours and relevant institutions, and share information with them as necessary.	2.76	(0.71)	2.05	3.48	0.634**
Q17.	I discuss a care plan with the person with dementia, their families and relevant stakeholders, provide decision‐making support as necessary, and propose a care plan that suits the preferred lifestyle of person with dementia.	3.01	(0.61)	2.40	3.61	0.678**
Q18.	I recommend the use of medical social welfare services outside the long‐term care insurance system (e.g. medical payment for services and supports for persons with disabilities, disability pensions, sickness and injury allowance, etc.) and informal services as necessary.	2.70	(0.73)	1.97	3.42	0.597**

^†^ Spearman's rank correlation coefficient.

** *P* < 0.01.

Next, the factor analysis of the 18 items was conducted and four factors were extracted with reference to scree plots (Table 3). All factor loadings were above 0.35 and four factors with eigenvalues of almost 1.0 or more were employed. The four factors were named Factor I, ‘Person‐centred care’; Factor II, ‘Understanding of disease characteristics, treatment and care’; Factor III, ‘Understanding of people with dementia and care management according to their characteristics’; and Factor IV, ‘Utilization of local resources surrounding people with dementia’.

**Table 3:** Component matrix after rotation of the scale

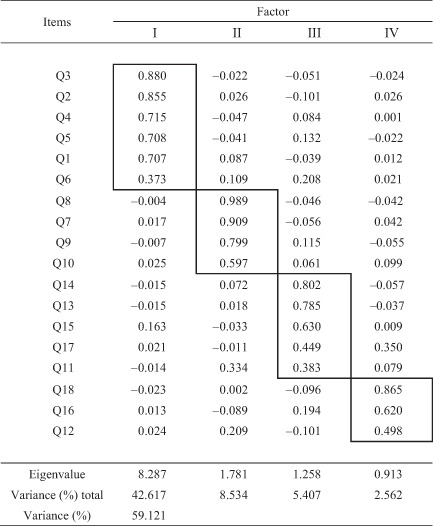

Furthermore, regarding the known‐groups method, we compared the four factor scores of the certified status and non‐certified groups (Table 4). The results showed that the group with certified chief care manager status scored significantly higher than the non‐certified group on Factors I (*P* = 0.013) and III (*P* = 0.026). Factors II and IV showed no significant difference between the two groups (*P* = 0.640 and *P* = 0.131, respectively). Supplemental File 2, in Data **S2**, shows the comparisons of the certified status and non‐certified groups, each further broken down for having versus not having and a medical qualification. There were no statistically significant differences between the groups thus defined with and without a medical qualification in any of the factors.

**Table 4:** Comparison of the groups with and without status as a certified chief care manager

Factors	Certified status group				Non‐certified group				*P*‐Value
	*n*	Mean	±	SD	*n*	Mean	±	SD	
I. Person‐centred care	383	20.3	±	2.9	241	19.8	±	2.5	0.013
II. Understanding of disease characteristics, treatment and care	382	11.8	±	2.2	239	11.7	±	2.0	0.640
III. Understanding of people with dementia and care management according to their characteristics	386	15.6	±	2.4	237	15.2	±	2.2	0.026
IV. Utilization of local resources surrounding people with dementia	384	8.3	±	1.8	242	8.1	±	1.6	0.131

*Note*: Student's *t*‐test.

Correlation coefficients between the four subscales were calculated and showed significant positive correlations with each other (0.453–0.663, *P* < 0.01). Cronbach's alpha coefficients, which assess internal consistency, were >0.7 for all four subscale factors (Table 5).

**Table 5:** Cronbach's alpha coefficients of the factors and inter‐factor correlation coefficients

Factors	I	II	III	IV	Total
Cronbach's alpha coefficient					
	0.879	0.908	0.848	0.716	0.928
Inter‐factor correlation coefficients^†^					
II	0.533**				
III	0.650**	0.663**			
IV	0.453**	0.524**	0.620**		

^†^ Pearson's correlation coefficient.

** *P* < 0.01.

## DISCUSSION

The purpose of this study was to develop a self‐assessment scale for the care management of people with dementia living at home. The four‐factor scale measured the concept well, with moderate to high correlation coefficients between subscales. For known‐groups validity, statistically significant differences were found for Factors I and III, while Factors II and IV did not differ depending on whether or not the participants were certified as chief care managers. Cronbach's alpha coefficients for the four factors and all items were greater than 0.7, which demonstrated good internal consistency.^22^

Against the backdrop of the increasing care needs of people with dementia living at home, care managers' reflection using this scale could lead to improved care management. In the factor analysis, the eigenvalue of Factor I, ‘Person‐centred care’, was the highest. In the home setting, the emphasis is on individualized care, based on the conditions, needs, preferences, and available resources for people with dementia.^23^, ^24^, ^25^ It was considered that care management for home‐dwelling people with dementia should focus on helping them continue living in their community, while promoting their autonomy^26^ and abilities and getting them to participate in care.^27^ It was reported that caregivers have tried to work to emphasize person‐centred care based on their competencies even in crisis situations under the COVID‐19 pandemic.^28^ When it was difficult to continue usual dementia care during the pandemic, they discussed care priorities with stakeholders and supported decision‐making in care. This competency is the foundation of dementia care management. In addition, the scores of those who were certified as chief care managers tended to be higher than those of those who were not certified in Factors I and III. Chief care managers have sufficient knowledge and skills in care management and are specially trained in dementia and care management. Therefore, it was corroborated that there is a certain relationship between higher scale scores and greater knowledge, skills, and attitudes in the care management of people with dementia, although this study is a cross‐sectional survey. However, this is the subjective measure. Future research is needed to find clinically significant changes of the extent to which changes in scores on this scale can be regarded as an improvement in care management competence.^29^

In contrast, Factors II and IV did not differ depending on whether the participants were certified as chief care managers or not. This may be due to the fact that many of the participants in this study had relatively long years of experience and there was no difference in years of experience between the groups with and without status as a certified chief care manager. Thus, they had continuously learned and updated their medical and long‐term care knowledge related to dementia and care resources. Recently, a significant proportion of households with people with dementia are instances of a person with dementia living alone.^30^, ^31^ The care needs of people with dementia have become increasingly complex, such as cases with difficulties in obtaining family support and cases with high medical dependencies.^32^ In addition, knowledge relevant to dementia treatment and other related medicine and care, and to support systems for people with dementia, is continually updated over time. Learning complex content hinders effective learning and does not improve motivation if it is self‐taught, such as e‐learning or reading textbooks, or if the content is not role‐appropriate.^33^ Therefore, when designing learning support for the care management of people with dementia that includes these elements, the content should be based on the role of care managers. In addition to basic knowledge, it should include specific situations where practical application is possible. Interactive learning and appropriate feedback are also highly useful to facilitate greater learning.^34^ In the future, it will be necessary to design learning programs that meet the needs of the learners, as well as to further verify whether this scale adequately assesses the knowledge of those being evaluated. Along with the validation of change through educational intervention, it may be desirable to use a combination of knowledge and skill tests to validate the change.

However, with this confirmation of a certain degree of validity and reliability for the scale, the researchers consider that it can be utilized by and for care managers to reflect on their practice in clinical settings as well as in educational interventions.

This study has several limitations. First, the study population was limited to care managers who belonged to a single association in a particular region. Future studies should expand to include participants in multiple regions and compare them with the results of this study. Furthermore, since this study was a cross‐sectional survey, it could not be verified whether this scale can adequately capture changes in the self‐evaluation scores of care managers with respect to their care management. Second, since this is a self‐assessment scale, it is not possible to assess the relationship between the scores and the actual knowledge, skills, and attitudes toward care management. It should be noted that while change within a single care manager may be measured, comparisons across multiple care managers are not possible.

## CONCLUSIONS

In the present study, we defined the competencies required for care management for people with dementia and developed a four‐factor scale with 18 items. The scale demonstrated good construct and criterion validity and internal consistency. This scale can clarify the content of care management based on person‐centred dementia care approach for people with dementia and help care managers reflect on their practice. Future research employing samples in various regions should confirm clinically significant changes in this scale.

## AUTHOR CONTRIBUTIONS

Kazawa and Ishii were involved in research design, analysis, and manuscript writing. Mochizuki and Ochikubo were involved in interpreting the data and manuscript writing. All the authors read and approved the final manuscript.

## DISCLOSURE

The authors received no financial support for the research, authorship, and/or publication of this article. The authors declare that they have no conflicts of interest directly relevant to the present study.

## Supporting information


**Data S1.** A self‐assessment scale for dementia care management, Japanese version

**Data S2.** Comparison of the groups with and without status as a certified chief care manager, and with or without medical qualification
PWD: people with dementia
The 114 were certified chief care managers and had medical qualification.
The 259 were certified chief care managers and did not have medical qualification.
The 60 were not certified chief care managers and had medical qualification.
The 173 were not certified chief care managers and did not have medical qualification.

## Data Availability

The datasets analyzed during the current study are not publicly available because this study was conducted as a project for the dementia special committee of Regional Health Care Council of Hiroshima Prefecture. No consent for secondary use of the data was obtained from the participants.
