# Supplementary material for: Development, reliability, and validity of a self‐assessment scale for dementia care management
Source: Psychogeriatrics. 2023 Feb 1;23(2):345–53. doi: 10.1111/psyg.12937 (PMC11577988; doi:10.1111/psyg.12937)
Supplement: Supplementary file 1 — Data S1. A self‐assessment scale for dementia care management, Japanese version [file PSYG-23-345-s002.pdf]

Supplemental File 1. A self-assessment scale for dementia care management Japanese version

	項目
Q 1	認知障害に関する行動には，まず本人の視点でその行動の理由を理解しようとする
Q 2	「なにもできない」とあきらめず、本人の視点で「なにかができる」と感じられるように支援することを心掛けている
Q 3	個人の独自性を尊重し、認知機能と本人に合わせて支援することを心掛けている
Q 4	認知症症状の軽重にかかわらず、本人の意思や価値を尊重して支援することを心掛けている
Q 5	本人の独自のニーズや不安を受け止めようとしている
Q 6	転倒のリスクなど起こり得る問題を予測した上で、常に本人にとって何が優先されるのか検討している
Q 7	認知症の中核症状および必要なケアについて理解している
Q 8	認知症の行動・心理症状および必要なケアについて理解している
Q 9	認知症の各進行段階における症状および必要なケアについて理解している
Q 10	認知症を起こす個々の疾患について概念や特徴、治療について理解している
Q 11	認知症の人や家族に生じる様々な身体的、心理的・社会的ニーズについて理解している
Q 12	成年後見制度や運転免許返納等認知症に関わる制度について理解している
Q 13	本人・家族等の意思、認知症の人の心身の状況や家族に介護状況などを把握できる
Q 14	把握した情報から、認知症の人にとって必要なケアとその優先順位を検討できる
Q 15	介護支援専門員として、認知症の人や家族・支援者が抱く感情や思いによりそう事が出来る
Q 16	近隣住民や関係機関等と連携する体制を構築し、必要に応じて情報を共有できる
Q 17	本人、家族、関係者とともに話し合い、必要に応じて意思決定支援を行い、本人が望む暮らしに沿ったケアプランを提案できる
Q 18	必要に応じて介護保険制度以外の医療社会福祉サービス（自立支援医療、障害年金、傷病手当等)、インフォーマルサービスの利用を提案できる
