# Supplementary figures and images for: Development, reliability, and validity of a self‐assessment scale for dementia care management
Source: Psychogeriatrics. 2023 Feb 1;23(2):345–53. doi: 10.1111/psyg.12937 (PMC11577988; doi:10.1111/psyg.12937)

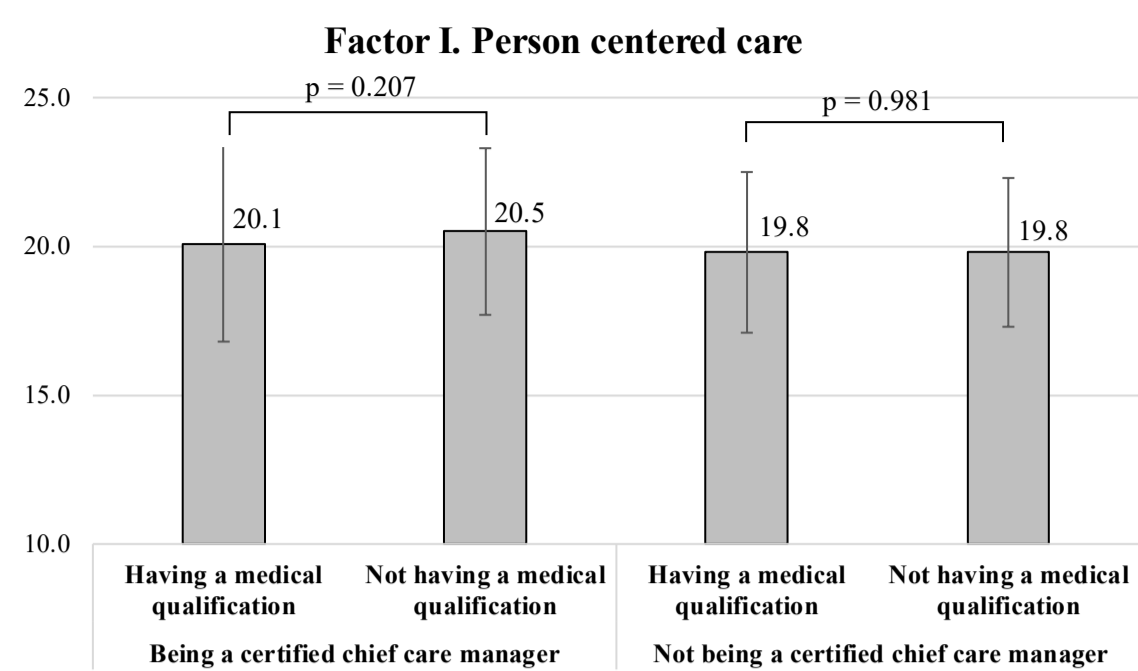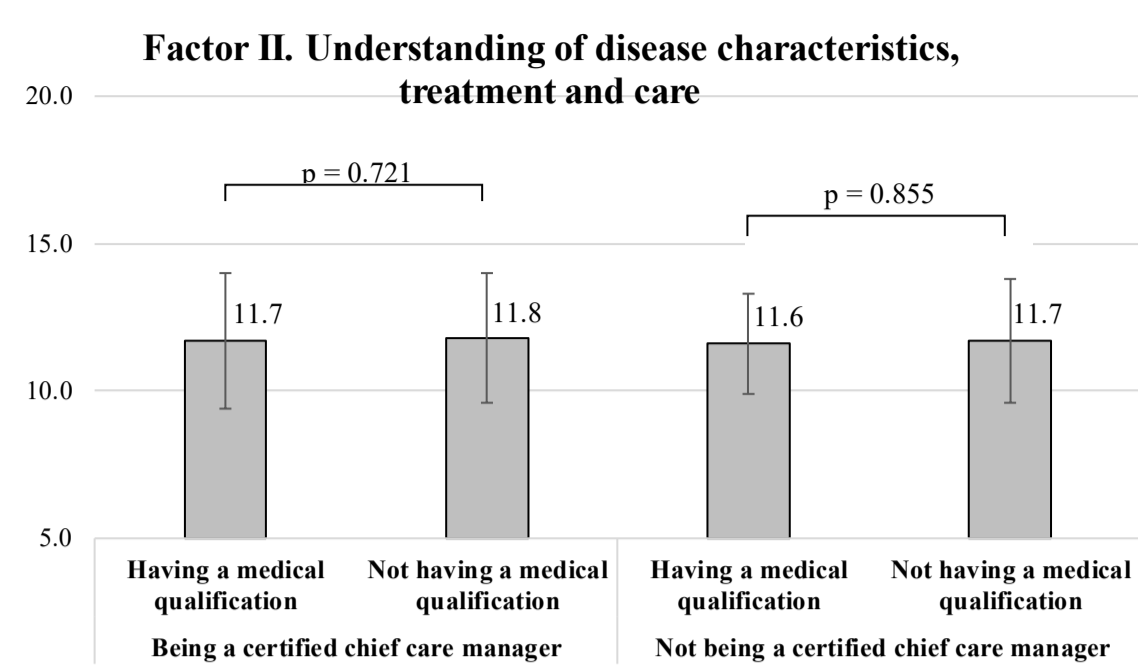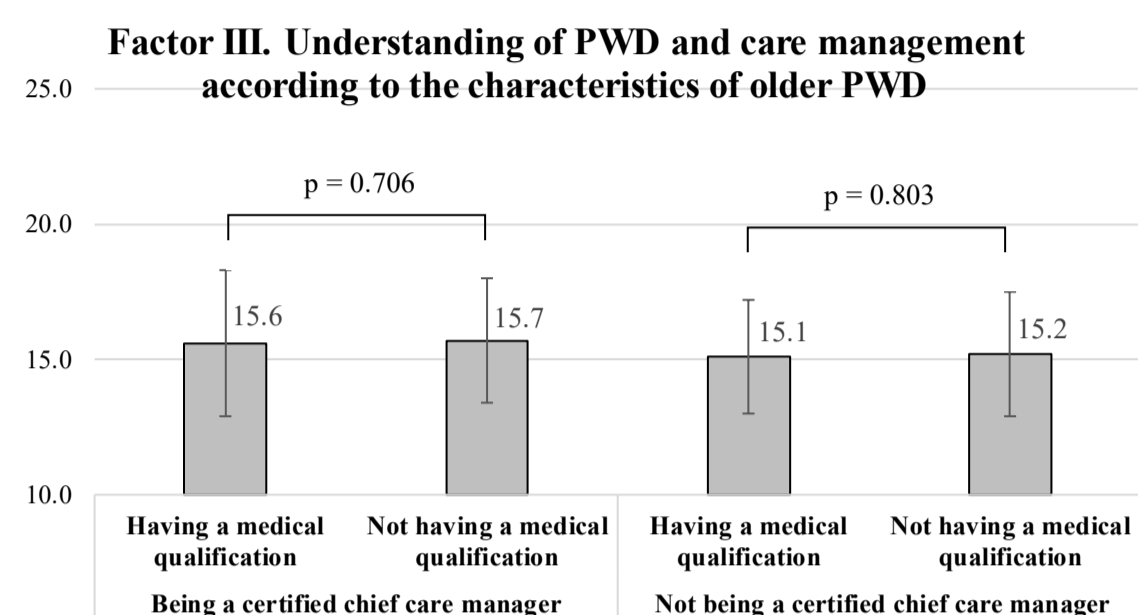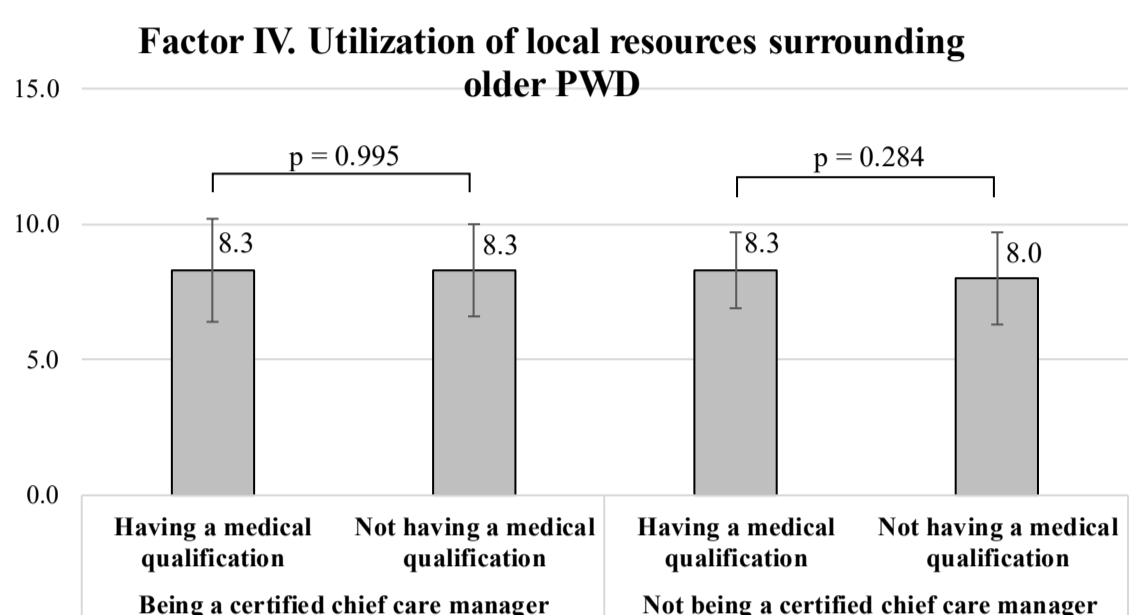

Supplement: Supplementary file 2 — Data S2. Comparison of the groups with and without status as a certified chief care manager, and with or without medical qualification PWD: people with dementia The 114 were certified chief care managers and had medical qualification. The 259 were certified chief care managers and did not have medical qualification. The 60 were not certified chief care managers and had medical qualification. The 173 were not certified chief care managers and did not have medical qualification. [file PSYG-23-345-s001.pdf]
